# Smartphone learning as an adjunct to vascular teaching – a pilot project

**DOI:** 10.1186/s12909-018-1148-8

**Published:** 2018-03-15

**Authors:** Nadeem A. Mughal, Eleanor R. Atkins, Darren Morrow, Wissam Al-Jundi

**Affiliations:** 1 Department of Vascular Surgery, Norfolk and Norwich University Hospital, Colney Lane, Norwich, UK; 2 Department of Vascular and Endovascular Surgery, The Royal Oldham Hospital, Pennine Acute Hospitals NHS Trust, Manchester, UK

**Keywords:** M-learning, Vascular surgery, Smartphones, Teaching adjuncts, MCQs

## Abstract

**Background:**

M-learning is education using personal mobile electronic devices. Given the prevalence of these in society and amongst healthcare professionals, we aimed to assess their use and feasibility in improving the educational programme of a single vascular institution.

**Methods:**

A weekly vascular departmental teaching programme was initiated with registrars giving 30-min presentations on a defined book chapter. Two multiple-choice questions (MCQ) per session were devised by a supervising consultant utilising the smartphone response system application, Polltogo. A separate investigator disseminated one pre-teaching and one post-teaching MCQ to the attending trainees via a WhatsApp group. Instant feedback of the correct answer was provided by the application. Participants’ satisfaction was judged through a survey after 13 sessions.

**Results:**

11 junior doctors of varying seniority participated in the trial. The median number of session attendees was 5. 129 MCQ responses were received. The mobile engagement score (number of answers received divided by total possible answers) was 97.7%. The average correct score for pre-teaching MCQs was 39.4% and post-teaching MCQs 73.0% (*p* < 0.001). Satisfaction with the concept was high; 80% of responders agreed that it was a useful adjunct to the teaching programme whilst 90% found the system highly user-friendly.

**Conclusions:**

Smartphones can be utilised effectively and with high user satisfaction in assessing knowledge transfer throughout a departmental education programme. Trainees’ responses to MCQs significantly improved after 30-min teaching sessions. This concept of m-learning could be developed further to assist with postgraduate examination revision or Deanery teaching programmes in larger cohorts.

## Background

In 2017, the communication market report indicated that up to 94% of adults in the United Kingdom possess a mobile phone [1]. Of these, 76% own a smartphone. The New Media Consortium Horizon Report in 2016 emphasised the increasing prevalence of BYOT [bring your own technology) in higher education [2]. BYOT refers to the practice of people bringing their own laptops, tablets, smartphones, or other portable devices with them to learning or work environments. In addition to surfing the Internet, smartphones possess the ability of loading various applications that can be utilised for entertainment and education.

Mobile learning (m-learning), defined as learning that takes place via a mobile electronic device, enables education without the traditional restrictions of static electronic or paper-based media [3]. Several m-learning schemes were found to be associated with high acceptance rates among undergraduates [4, 5]. While electronic learning (e-learning) has been widely utilised for education purposes over many years, m-learning schemes are now becoming increasingly common with the advent of more portable and technologically advanced mobile devices. These devices enable access to key facts at the ‘point-of-need’ and allow use to be made of otherwise wasted time throughout the day. Such immediate access to information aids the consolidation of knowledge [6]. In addition to this access to knowledge, previous systematic reviews demonstrated that mobile devices enhance learner engagement and can provide an instant means of assessment and feedback [7, 8].

Within medical education, the application of smartphones has been rapidly expanding. In addition to online resources and podcasts, smartphones allow access to medical textbooks for clinical referencing. There are also approximately 10,275 applications available on various online stores ranging from medical calculators and flash cards to laboratory tests and radiology tutorials [9]. Several pedagogical learning theories have been associated with m-learning, including the behaviorist theory. The behaviorist-learning paradigm is based on learning through the reinforcement of an association between a particular stimulus and a response [10]. Such a drill and feedback model can be applied to m-learning through the presentation of a problem or content specific question (stimulus). The learner then contributes through a solution (response) and the system finally provides feedback (reinforcement).

A classic example within m-learning would be a response system. Such an audience response (AR) system is increasingly used at educational institutions worldwide in order to foster interactive learning and student engagement, in particular with large student numbers [11]. AR systems typically consist of a hardware transmitter (“clicker”) that is controlled by the student, a radiofrequency receiver and a computer with software to display voting results during a presentation by the instructor [12]. AR systems are used in a variety of ways: to increase student interaction and attention, to promote in-class discussions, to evaluate student knowledge or as formative assessments for the lecturer to adjust teaching pace and didactics [11]. Recent meta-analyses in health profession education showed that AR systems are favoured by the vast majority of students and are likely to result in improved short-term and long-term knowledge outcomes [13].

Smartphones are perfectly suited for such an application as multiple choice questions (MCQs) can be disseminated to a group of students, allowing them to answer the questions when feasible, receive electronic feedback and discuss the topics on an online forum. To test this, we have conducted a study that aims to assess the use and feasibility of smartphone education in improving the educational programme of a single vascular institution, utilising a response system application with immediate directive e-feedback.

## Methods

**Fig. 1:**
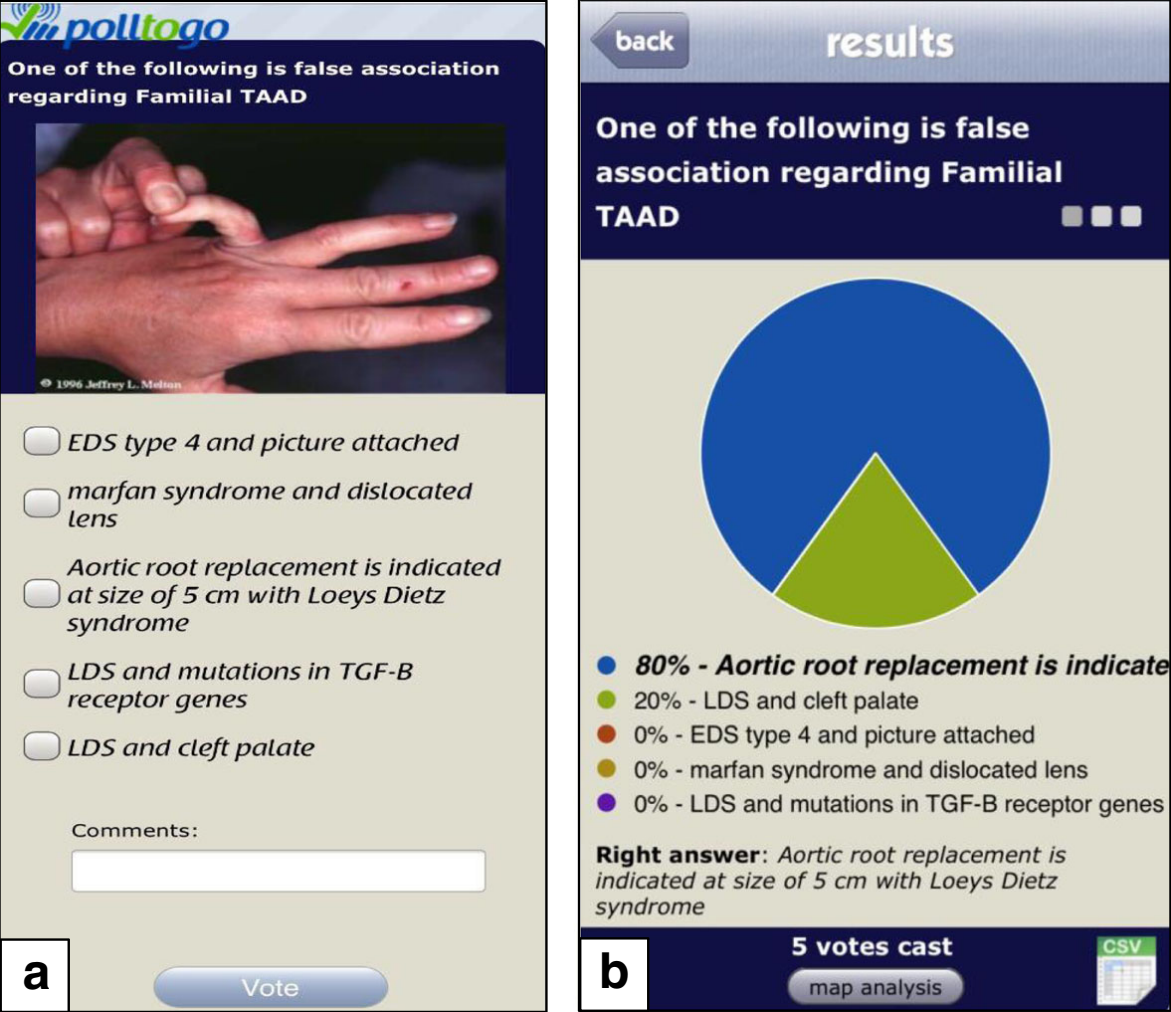
(**a**) Example of an MCQ question with (**b**) the instant feedback provided on answering

This study was performed at the Norfolk and Norwich University Hospital between April and September 2017. The vascular department comprised 12 junior doctors, all of whom owned a smartphone and used the messaging service WhatsApp. A weekly teaching programme was initiated with trainees giving 30-min sessions on pre-defined textbook chapters. Prior to each session, two questions relating to the chapter were devised by a supervising consultant (investigator 1) using a smartphone response system application (Polltogo, Inspirapps Inc.). This platform allows the user to create questions in either an MCQ or true/false format with supplementary images included if required (Fig. 1). Only one attempt at the question is possible and on answering, immediate feedback of the correct answer is given as well as an anonymised spread of other participant responses.

A WhatsApp chat group named ‘Vascular Teaching’ was setup and all trainees were invited to join. Each week a separate investigator (investigator 2) randomly disseminated one of these MCQs to the attendees at the start of the session and one at the end via a link on the WhatsApp chat group. Clicking on this link took the user straight to the question on a separate webpage. The level of attending trainees, the mobile engagement score (number of answers received divided by number of possible answers) and anonymised pre- and post-teaching scores were recorded by investigator 2. At the end of the study a short questionnaire was sent out to gain feedback on participant satisfaction with the m-learning experience (Table 1). Statistical analysis was performed using the Statistical Package for the Social Sciences (SPSS) version 22.0 (IBM Corp, Armonk, New York). The Chi squared test was used to assess for differences in categorical variables with a *p* value < 0.05 considered significant and < 0.001 highly significant. Participants entered the study on a voluntary basis and provided written informed consent prior to it commencing. The National Health Service Health Research Authority confirmed that no ethical approval was required for this study.

**Table 1:** Survey sent to participants at the end of the study period

	**Question**
**1**	Did you find the Smartphone MCQs useful in the teaching programme?
**2**	Would you recommend their continued use?
**3**	Is the setup of WhatsApp messages with links to the question easy to use?
**4**	Do you think more MCQs would be a good adjunct to revision for postgraduate exams?
**5**	Do you think the pre and post MCQ questions were of equal difficulty?
**6**	Were the questions suitable for your level of training?
**7**	Was there anything particularly good?
**8**	Was there anything that could be improved?

## Results

**Fig. 2:**
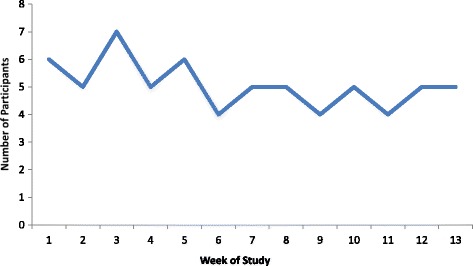
Number of participants attending weekly teaching sessions throughout the study period

**Fig. 3:**
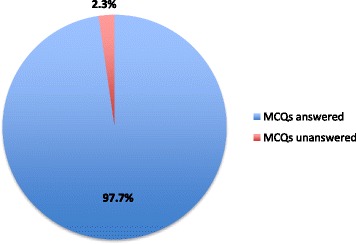
Percentage of available MCQs answered throughout the study (mobile engagement score)

**Fig. 4:**
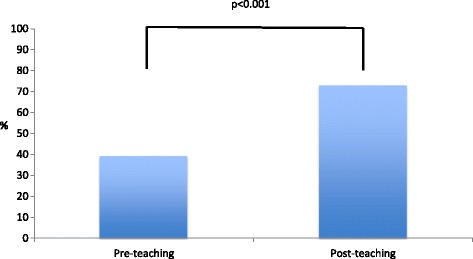
Comparison of pre- and post-teaching MCQ scores

11 of the 12 (91.6%) departmental junior doctors participated in the study when rota and leave commitments allowed. One was unable to make the sessions due to personal circumstances. The group comprised 4 foundation trainees, 2 core surgical trainees, 3 specialty trainees, and 2 staff grade registrars. A pre-study survey confirmed that each participant possessed a smartphone. The spread of attendees throughout the sessions is shown in Fig. 2. The median number of session attendees was 5 (interquartile range 4.5–5.5). In total, 129 MCQ responses were received out of a possible 132, giving a mobile engagement score of 97.7% (Fig. 3). The average correct score for pre-teaching MCQs was 39.4% (26/66) and for post-teaching MCQs 73.0% (46/63), representing a highly significant improvement (*p* < 0.001). These results are outlined in Fig. 4.

**Fig. 5:**
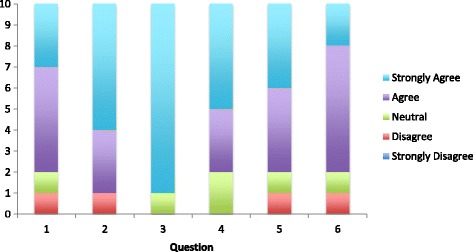
Breakdown of participant satisfaction with smartphone learning

10 of 11 (90.9%) participating junior doctors filled in the survey at the end of the trial (Fig. 5). 80% of respondents either ‘agreed’ or ‘strongly agreed’ that the MCQs were useful in the teaching programme and 90% recommended their continued use. 90% ‘strongly agreed’ that the setup was easy to use and 80% believed that MCQs would be a good adjunct to revision for postgraduate specialty collegiate exams. The last two boxes of the survey were for free text comments and the results of these are shown in Table 2.

**Table 2:** Participant free-text feedback on end of study survey

**Good points**	**Improvements**
‘Excellent to consolidate learning from sessions’	‘Maybe more time to answer questions’
‘Immediate visual feedback of correct answers useful’	‘A batch of 3 pre- and post- questions per session would consolidate learning better’
‘Easy and quick’	‘More than 1 question per session needed to reflect knowledge gained’
‘Innovative method of learning’	
‘Sessions are helpful for exam revision’	
‘Very easy to use’	
‘Works well to consolidate knowledge’	
‘Very focused and quick way to learn’	
‘A good way to test the learning of new material’	

## Discussion

Smartphones and tablets are changing the way medicine is taught and practised [14, 15]. Studies show that more than 92% of healthcare professionals, including medical students, residents, and supervising physicians, utilise smartphones or tablets in patient care-related activities [16]. The simplest example is surfing the Internet to retrieve medical information. Other uses include reading textbooks, browsing surgical techniques, referencing drugs, filling clinical logbooks, and researching upcoming conferences and events [17–19].

There is considerable interest from educators and technical developers in exploiting the unique capabilities and characteristics of mobile technologies to enable new and engaging forms of learning. Some of the perceived advantages of using smartphones include learning anywhere and anytime, offering unlimited capacity since there is no need for a classroom. Hence, smartphone-based education can provide a self-directed learning environment that allows users to repeatedly access information and practice skills without any space and time limitations. Such factors and mobility in learning can enhance autonomy, self-efficacy and students’ engagement, factors that have been associated with better learning outcomes [20–22]. Moreover, using electronic technology in health-care delivery and education is cost-effective and allows immediate online information sharing including text, pictures and videos [23].

Clearly a pre-requisite for m-learning is user possession of a smartphone. A recent survey amongst orthopaedic trainees and surgeons revealed that 97.7% were using smartphones, while all the trainees in our study did so [24]. Importantly, our cohort was happy to use them for this adjunct to teaching and the vast majority found the setup highly user-friendly.

Teaching postgraduate trainees is effectively an attempt to improve their knowledge, skills and attitude. The importance of knowledge in clinical practice cannot be underestimated. However, in order to achieve a deep level of learning, it is insufficient to simply know the facts and have the knowledge; rather, developing the ability to understand and correctly apply the knowledge is needed. Active participation of the learner through a process of assessments and feedback is vitally important in achieving this understanding according to cognitive psychology research [25]. This process stimulates a pedagogic active learning approach that helps in improving the depth and breadth of learning [26]. In addition, repeated educational activities supported with feedback have been proven to aid retention of knowledge as well, compared to simply studying before a final exam [27]. We theorised that pre- and post-teaching MCQs would not only confirm the efficacy of the teaching, but also attempt to consolidate the learner’s knowledge by putting it into practice. The teaching programme was proven to be effective with a jump in MCQ scores from 39.4% to 73% following the sessions. Feedback from learners was highly positive in terms of the usefulness and ease of access, and this was shown quantitatively through the mobile engagement score of 97.7%. Two survey responders mentioned that a single MCQ question might not be enough to gauge the effectiveness of the teaching programme and multiple MCQs could consolidate learning better. This was noted by the authors and should be considered in further uses of the technology. The results of this study showed that a single question adequately reflected knowledge gained and fit with the time constraints of a short pre-ward round tutorial. Additional questions could be added in but this would require additional time at the beginning and end of each session and might be viewed more as testing/examining rather than teaching.

Limitations of this study include its short period of time and small number of participants. It did however involve 11 of 12 departmental junior doctors and the positive feedback received has led to m-learning becoming a regular activity planned for the foreseeable future. Continuous data collection and periodical surveys will show whether efficacy and satisfaction remain high in the long-term. There is certainly the potential to expand this method of learning to other hospital departments or larger groups including cohorts sitting postgraduate specialty collegiate exams.

Another pre-requisite for a successful programme is ready access to the Internet. In our institution there is a free Wi-Fi network but many hospitals do not have these and other rapid data connections such as 3G/4G are not universally available. One technical limitation noticed during the study was the character limit for MCQ answers. Whilst the Polltogo application allows up to 10 options for each question, these can only be 80 characters long, meaning more detailed answers are not possible. The investigator charged with devising MCQs managed to overcome this challenge relatively easily using succinct question design.

The application also only allows 20 free responses for each question before a financial charge is levied for any further participants.

## Conclusions

In summary, m-learning provides a means to modernise departmental education using widely available technology at no additional cost. Our study shows that smartphones can be utilised effectively and with high user satisfaction in assessing knowledge improvement throughout a departmental education programme. Trainees’ responses to MCQs significantly improved after 30-min teaching sessions and uptake of the concept as judged by mobile engagement score was very good. As more people use smartphones and the number of applications to facilitate knowledge transfer grow, the potential for use in medical education and clinical practice will continue to increase. At a local level this could potentially be developed further to assist with postgraduate exam revision or Deanery teaching programmes in a larger cohort.

## Abbreviations

AR: Audience response
BYOT: Bring your own technology
E-learning: Electronic learning
MCQ: Multiple choice question
M-learning: Mobile-learning
SPSS: Statistical package for the social sciences
